# Genetic Diversity and Ecological Distribution of *Bulinus* Populations in Southern Benin Using PCR‐RFLP Analysis

**DOI:** 10.1155/bmri/5536391

**Published:** 2026-03-25

**Authors:** Elokou Alabi, Aboudou Chabi Do, Jonathan Nougbode, Halfane Lehmane, Haziz Sina, Paulin Sedah, Adolphe Adjanohoun, Michel Sezonlin, Lamine Baba-Moussa

**Affiliations:** ^1^ Research Unit in Phylogeography, Genetics and Evolution, Faculty of Science and Technology, University of Abomey-Calavi, Abomey-Calavi, Benin, uac.bj; ^2^ Laboratory of Biology and Molecular Typing in Microbiology, Faculty of Science and Technology, University of Abomey-Calavi, Abomey-Calavi, Benin, uac.bj; ^3^ Laboratory of Molecular Genetics and Genome Analysis, University of Abomey-Calavi, Abomey-Calavi, Benin, uac.bj

**Keywords:** Benin, *Bulinus forskalii*, *Bulinus globosus*, *Bulinus truncatus*, *Cox1*, molecular diversity, PCR-RFLP, schistosomiasis, *SSU*

## Abstract

Schistosomiasis remains a major public health concern in Benin. Freshwater snails of the genus *Bulinus* serve as intermediate hosts for schistosomes, trematode parasites responsible for bilharzia. The urinary form, caused by *Schistosoma haematobium*, is the most prevalent and is primarily transmitted by *Bulinus truncatus* and *Bulinus globosus*, whereas the intestinal form, caused by *Schistosoma mansoni*, is transmitted by *Bulinus forskalii*. Effective control strategies require an understanding of the molecular diversity and genetic structure of *Bulinus* populations. *Bulinus* samples were collected from six localities in southern Benin: Sô‐Ava, Azowlissè, Aplahoué, Kpinnou, Houéyogbé, and Za‐Kpota. Samples were preserved in absolute alcohol. DNA was extracted, and fragments of the *Cox1* and *SSU* genes were amplified. Molecular diversity was assessed using PCR‐RFLP, with amplified products digested using *HaeIII*, *HinfI*, and *DdeI* restriction enzymes. Molecular variance analysis (AMOVA) was performed to evaluate population structure. AMOVA revealed heterozygosity deficits in *Bulinus* populations from all six localities and deviations from panmixia in Sô‐Ava, Aplahoué, Houéyogbé, Kpinnou, and Za‐Kpota. Enzymatic digestion indicated monomorphism for *B. globosus* in Za‐Kpota and Houéyogbé with the *SSU* gene and for *B. truncatus* in Sô‐Ava and Azowlissè with the *Cox1* gene. Conversely, molecular polymorphism was observed for *B. globosus* in Sô‐Ava and Aplahoué with the C*ox1* gene, whereas *B. truncatus* in Sô‐Ava and Azowlissè showed identical *SSU* gene profiles. These findings provide preliminary insights into the molecular diversity and genetic structure of *Bulinus* populations in southern Benin. The observed genetic variability and structure have implications for understanding the transmission dynamics of *S. haematobium* and for designing targeted schistosomiasis control strategies.

## 1. Introduction

Human schistosomiasis is an acute and chronic disease often overlooked in tropical regions. Blood flukes of the *Schistosoma* genus, also known as schistosomes, cause it. Following malaria, schistosomiasis ranks as the second most prevalent parasitic disease globally [1]. According to the World Health Organization (WHO), there is an apparent mismatch between the disease′s prevalence and the healthcare resources allocated to combat it. For instance, in 2021, 251.4 million people required preventive treatment for schistosomiasis, but only 75.3 million received treatment. Schistosomiasis transmission has been documented in 78 countries [2]. In 2021, the COVID‐19 pandemic and efforts to mitigate its impacts resulted in decreased interventions against neglected tropical diseases (NTDs). Despite being a significant public health issue, these parasitic diseases are often classified as neglected due to the persistent focus on more prominent health challenges such as HIV, tuberculosis, and malaria [3]. Epidemiological data indicate that the number of people affected by schistosomiasis has not significantly declined, despite the increased attention in recent years [3]. Schistosomiasis manifests in two primary forms: hepatointestinal and urogenital. Schistosome parasites depend on freshwater snails as intermediate hosts to complete the asexual stage of their life cycle, after which cercariae are released into water bodies to infect human hosts. Once inside humans, the parasites continue and complete their sexual development [4]. Although more than 20 *Schistosoma* species are recognized, only *Schistosoma mansoni* and *Schistosoma haematobium* are prevalent in most sub‐Saharan African countries, causing intestinal and urogenital schistosomiasis, respectively [5]. Among these, *S. haematobium* is the most widespread, infecting 112 million African people [6]. Poor hygiene and specific lifestyle practices, such as swimming or fishing in infested waters, make communities living near aquatic environments, particularly school‐aged children, vulnerable to infestation [7]. The disease is most prevalent among children aged 0–18 years [8]. Infected human hosts generally exhibit acute hyperemia, abnormal growth, internal bleeding, tissue fibrosis and thickening, bladder cancer, and, notably, hypertrophic and ulcerative lesions in women′s genital organs, as well as liver fibrosis [9].

Most studies on *Bulinus* in Benin have focused either on the ecology of these intermediate snail hosts of schistosomiasis in relation to water quality and snail dynamics in the lacustrine areas of Sô‐Ava, southern Benin [10], or on the epidemiology of urinary schistosomiasis and geohelminthiasis among schoolchildren in the same administrative region [11]. Advances in molecular biology techniques, such as PCR‐RFLP markers [12, 13], RAPD [14], microsatellites [15, 16], and mitochondrial and ribosomal markers [17–20], have enhanced the understanding of molecular ecology and genetic diversity in *Bulinus* species populations. Studying genetic diversity and structure allows for the identification of populations and an understanding of their evolutionary forces. This study is aimed at examining molecular polymorphism through PCR‐RFLP in two gene fragments, determining the genetic composition of populations, determining populations′ genetic composition, and identifying the contributing factors. The conclusions drawn from this study will inform the development of tailored biological control strategies for each species and their respective populations, thereby establishing a foundation for the rational management of parasites and their hosts.

## 2. Materials and Methods

### 2.1. Study Area

**Figure 1:**
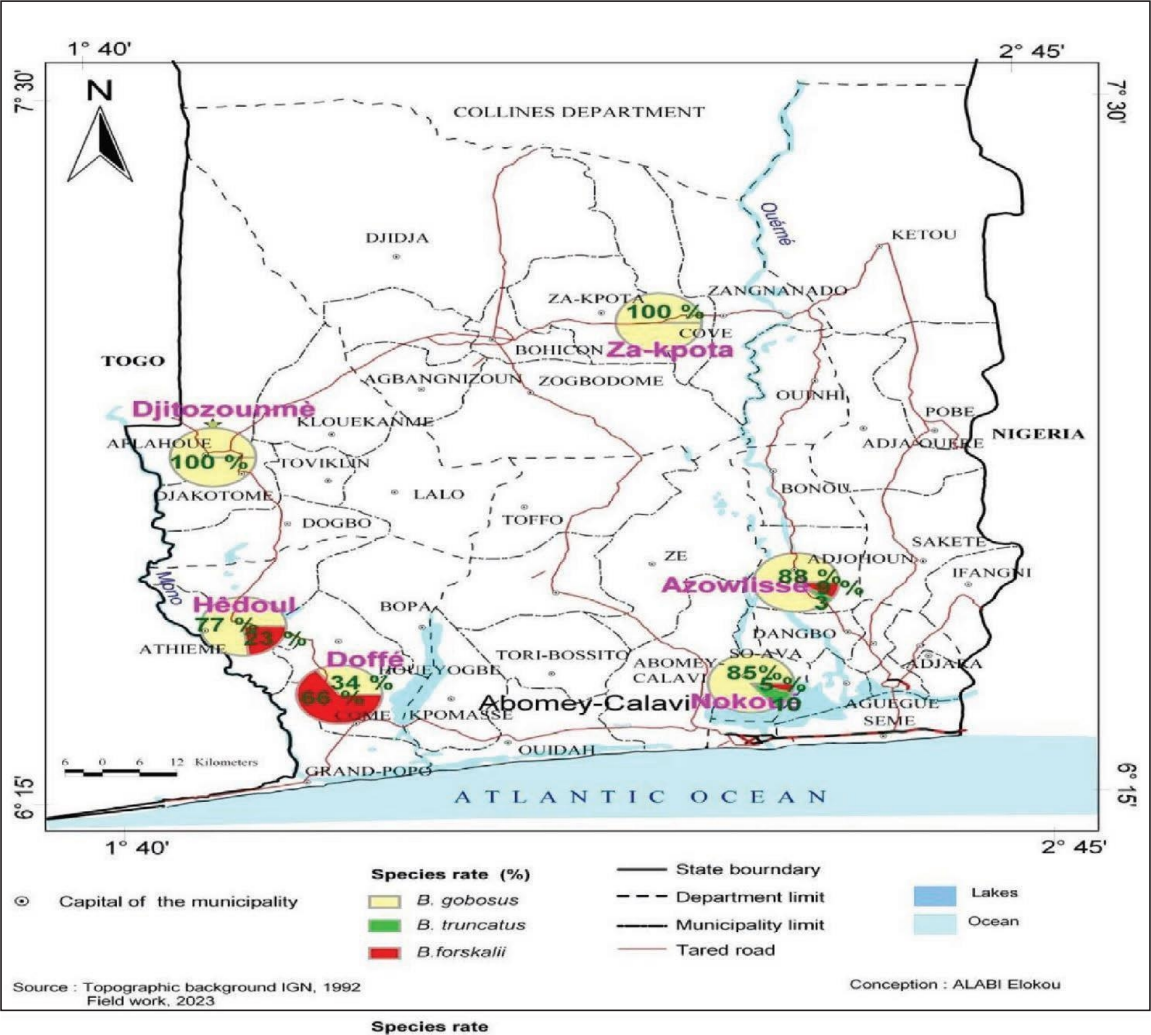
Geographical distribution of the different *Bulinus* species found in southern Benin.

This study was conducted between 2022 and 2023 in six localities across five departments in southern Benin (Figure 1): Sô‐Ava on Lake Nokoué (Atlantique), Djitozounmè in Aplahoué (Couffo), Doffé in Houéyogbé and Hêdoui in Kpinnou (Mono), Azowlissè in Adjohoun (Ouémé), and Za‐Kpota (Zou). Gastropods were collected over 2‐month periods each year: January–February 2022 and November–December 2023. Sampling was performed during the dry season to ensure comparable hydrological conditions across sites. All sites are located in southern Benin, characterized by a subequatorial climate with high humidity [21, 22].

### 2.2. Sampling and Sample Collections

This cross‐sectional malacological survey was conducted in southern Benin from May to October 2023, encompassing major freshwater ecosystems, including Lake Nokoué and Lake Ahémé, as well as the Ouémé, Mono, Couffo, and Zou river basins. These hydrosystems are characterized by frequent human–water interactions associated with irrigated agriculture, fishing, aquaculture, and domestic activities, thereby creating environments conducive to schistosomiasis transmission. Sampling sites were selected based on predefined hydrographic and anthropoecological criteria. Specific collection points were determined through prior ecological assessments of lentic and semilotic habitats suitable for pulmonate snails, complemented by an exploratory community survey to identify water contact sites frequently used by local populations (laundry, bathing, fishing, and market gardening) [23]. This targeted approach ensured that sampling focused on epidemiologically relevant transmission hotspots. At each site, *Bulinus* snails were collected using a standardized active search protocol with a fixed sampling effort of 2 h. The collection encompassed multiple microhabitats including vegetated margins, shallow zones, and muddy or sandy substrates to maximize representativeness and mitigate spatial sampling bias. The georeferencing of the sampling sites was conducted using a Global Positioning System (GPS). A total of 407 snails were collected across six sites, with the sample size determined a priori according to established malacological field protocols to provide sufficient statistical power for both ecological and molecular analyses. Specimens were transported in perforated containers containing site water to the Phylogeography, Genetics, and Evolution Research Unit (UR‐PGE), Faculty of Science and Technology, University of Abomey‐Calavi, and processed on the same day. Morphological identification followed established taxonomic keys [8]: *Bulinus globosus* was distinguished by a truncated columella and shell microsculpture with nodules and short ridges; *Bulinus truncatus* by a straight, uniformly concave, or twisted columella without truncation; and *Bulinus forskalii* by a sharply pointed shell apex. The primary outcome was infection status, determined through cercarial shedding. Each snail was individually placed in a glass vial containing freshwater and exposed to artificial light for up to 4 h [24]. Cercarial emergence was monitored at regular intervals, and emerged cercariae were morphologically identified following established criteria [25] using a binocular microscope at 10× magnification. Following parasitological assessment, snails were counted and individually preserved in 70% ethanol for subsequent molecular analyses. To ensure methodological consistency and minimize both selection and measurement bias, standardized sampling durations, predefined ecological selection criteria, and uniform laboratory procedures were applied across all sites.

### 2.3. Molecular Characterization of Collected Samples

#### 2.3.1. DNA Extraction From *Bulinus* Snails

A total of 407 *Bulinus* snails were morphologically identified and collected. From these, a subset of infected individuals was randomly selected for molecular analysis, including 80 *B. globosus*, 10 *B. truncatus*, and 6 *B. forskalii*. To assess genetic diversity at each site, two to five snails per locality were randomly chosen [26]. Collected snails were preserved in absolute ethanol and transported to the Laboratory of Biology and Molecular Typing in Microbiology at the University of Abomey‐Calavi for DNA extraction. Tissue samples (20 mg, shell excluded) were rinsed in ethanol to remove mucus and homogenized. Samples were incubated sequentially in TE buffer and distilled water before treatment with preheated urea–SDS buffer and proteinase K, followed by mechanical disruption and overnight incubation for cell lysis. DNA was purified using phenol–chloroform–isoamyl alcohol extraction, precipitated with ethanol and sodium acetate, washed with 70% ethanol, air‐dried, and finally resuspended in 100 *μ*L ultrapure water. DNA quality and quantity were measured using a NanoDrop spectrophotometer, yielding a purity index (*I*) of 1.8–2.0, indicating optimal extraction.

#### 2.3.2. Amplification and Enzymatic Digestion of *Cox1* and *SSU* Genes

The amplification of the mitochondrial *Cox1* gene and the nuclear *SSU* (18S rRNA) gene was performed following the standard protocols described by [20, 27], with minor modifications. The [25] protocol employs universal primers (LCO1490 and HCO2198) to amplify an approximately 820 bp fragment of the *Cox1* gene, which is widely used for molecular barcoding and species identification. In contrast, the method developed by [20] targets a 1020 bp region of the *SSU* gene using specific primers designed for freshwater snails, providing improved phylogenetic and taxonomic resolution of *Bulinus* species (Table 1). In both protocols, PCR amplification followed a conventional cycling scheme comprising an initial denaturation, 35 amplification cycles (denaturation, annealing, and extension), and a final elongation step. The resulting amplicons were subsequently subjected to restriction enzyme digestion (restriction fragment length polymorphism [RFLP]) and electrophoretic analysis on agarose gels to assess genetic polymorphism among the samples.

**Table 1:** Characteristics of restriction enzymes used for the digestion of PCR‐amplified nucleotide fragments.

Regions analyzed	Enzymes	Nucleotide sequences	References
Mitochondrial DNA	*HaeIII*	5 ^′^ GG//CC 3 ^′^	[27]
		3 ^′^ CC//GG 5 ^′^	
ITS region	*DdeI*	5 ^′^ C↓TNAG 3 ^′^	[28]
		3 ^′^ GANT↑C 5 ^′^	
Mitochondrial DNA	*HinfI*	5 ^′^ G↓ANTC 3 ^′^	[29]
		3 ^′^ CTNA↑G 5 ^′^	

### 2.4. Statistical Analyses

Several methodological and analytical approaches were employed to comprehensively investigate the genetic structure of *Bulinus* populations sampled from various localities. Genetic and genotypic structures were analyzed using Genepop Version 4.2, which allowed estimation of allele frequencies at each hierarchical level based on the *Cox1* and *SSU* genes. Associated *p* values for each estimator were calculated using the binomial distribution implemented in R software Version 3.3.3, enabling validation of the results. Observed heterozygosity (*H*_*o*_) and expected heterozygosity *H*_*e*_, as well as the fixation index (*FIS*), which reflects the level of inbreeding within populations, were also calculated using R Version 3.3.3. These calculations followed the formulas described in [28, 29]: *H*_o_ was calculated as the ratio of heterozygous individuals to the total number of individuals in the sample. And *H*_e_ was derived from allele frequencies using the following formula: *H*_*e*_ = 1 − (*f*1^2^ + *f*2^2^ + *f*3^2^+⋯*f* *n* ^2^), where *f*1, *f*2, …, *f* *n* represent the frequencies of *n* alleles. RFLP data were coded as presence/absence (1/0) for each individual, generating a binary matrix used for all downstream analyses, including *FST* and AMOVA calculations. The Hardy–Weinberg equilibrium (HWE) test was performed using Genetics Version 1381 to evaluate the null hypothesis of panmictic populations. The *FIS* quantifies deviations from panmixia and is closely associated with the level of inbreeding within a population. It measures the deficit or excess of heterozygosity at a given locus within a subpopulation and is defined as follows: *F* *I* *S* = 1 indicates complete fixation, as observed in cases of self‐fertilization.

*F* *I* *S* > 0: Heterozygote deficit compared to panmictic equilibrium.

*F* *I* *S* = 0: Population in HWE.

*F* *I* *S* < 0: Excess heterozygosity.

Genetic differentiation (*FST*) is calculated within each population. This genetic divergence index reflects the decrease in heterozygosity related to divergence within the populations considered. Mathematically, *F* *S* *T* = (*H* *T* − *H* *S*)/*H* *T*, where *HT* represents the *H*_*e*_ of an individual in a total population assuming panmixia and *HS* corresponds to the *H*_*e*_ of an individual in a subpopulation assuming panmixia. Therefore, a parameter reflects genetic divergence within populations. If *FST* equals or is very close to 0, it indicates continuous and significant gene flow between populations. Conversely, if *FST* is close to 1, it means strong genetic differentiation between populations or reduced gene flow. The criteria defined by [30] were used to interpret the index. According to this author, genetic differentiation is considered low if the value is below 0.05 and moderate if the value falls between 0.05 and 0.15. It is considered substantial if the value is between 0.15 and 0.25 and very strong if it exceeds 0.25.

## 3. Results

### 3.1. Spatial Distribution and Specific Richness of *Bulinus* Species in Southern Benin

A total of 407 *Bulinus* specimens were collected across all sampling sites, comprising 338 *B. globosus* (83.0%), 23 *B. truncatus* (5.7%), and 46 *B. forskalii* (11.3%).

### 3.2. Abundance Rate of *Bulinus* Species in Different Localities

Table 2 presents the abundance rates of *Bulinus* species sampled across the different localities. Analysis of Table 3 revealed that *B. globosus* was present in all six study localities. The highest abundance was recorded at Sô‐Ava (50.30*%* ± 3.83*%*), while the lowest was observed at Kpinnou (2.96*%* ± 5.35*%*). Abundance rates in the other localities, in descending order, were as follows: Azowlissè (26.63*%* ± 4.65*%*), Aplahoué (10.35*%* ± 5.14*%*), Za‐Kpota (5.91*%* ± 5.27*%*), and Houéyogbé (3.84*%* ± 5.32*%*). *B. forskalii* was most abundant at Houéyogbé, with a rate of 54.35*%* ± 9.96*%*, higher than in other localities. *B. truncatus* was found in high abundance only in the two localities where it occurred, Sô‐Ava and Azowlissè, where it coexisted with the other two species.

**Table 2:** Specific richness of *Bulinus* species in various regions.

	Specific richness in various *Bulinus* species collected			
Species origin	*Bulinus globosus*	*Bulinus truncatus*	*Bulinus forskalii*	Total
Sô‐Ava	170	20	9	199
Azowlissè	90	3	9	102
Za‐Kpota	20	0	0	20
Aplahoué	35	0	0	35
Houéyogbé	13	0	25	38
Kpinnou	10	0	3	13
Total	338	23	46	407

**Table 3:** Abundance rate of *Bulinus* populations for the different species sampled across localities.

		Abundance rate of different *Bulinus* species populations			
	**Species**	***Bulinus globosus***	***Bulinus truncatus***	***Bulinus forskalii***	**Average abundance rate by locality (%)**
Provenance	Sô‐Ava	50.3 ± 3.83	86.96 ± 7.52	19.56 ± 13.21	52.27 ± 3.54
	Azowlissè	26.63 ± 4.65	13.04 ± 19.44	19.56 ± 13.22	19.74 ± 3.94
	Za‐Kpota	5.91 ± 5.27	0.00 ± 0.00	0 ± 0.0	1.97 ± 3.10
	Aplahoué	10.35 ± 5.14	0.00 ± 0.00	0 ± 0.0	3.45 ± 3.08
	Houéyogbé	3.84 ± 5.32	0.00 ± 0.00	54.35 ± 9.96	19.4 ± 6.41
	Kpinnou	2.96 ± 5.35	0.00 ± 0.00	6.53 ± 14.26	3.14 ± 4.83
	Average abundance rate of the host species (%)	16.67 ± 2.12	16.67 ± 7.77	16.67 ± 5.49	

**Table 4:** Infestation rates of *Bulinus* species in different localities.

	Infestation rate expressed as a percentage (%) of different species populations of *Bulinus* snails by schistosomes			
Origin	*Bulinus globosus*	*Bulinus truncatus*	*Bulinus forskalii*	Average infestation rate per area (%)
Sô‐Ava	64.12 ± 4.60	75.00 ± 11.18	66.67 ± 19.24	68.6 ± 4.07
Azowlissè	76 ± 4.90	66.67 ± 33.33	66.67 ± 19.24	69.78 ± 5.01
Za‐Kpota	100.00 ± 8.90	0.00 ± 0.00	0.00 ± 0.00	33.33 ± 10.54
Aplahoué	85.71 ± 6.39	0.00 ± 0.00	0.00 ± 0.00	28.57 ± 8.24
Houéyogbé	53.84 ± 18.84	0.00 ± 0.00	80.00 ± 8.94	44.61 ± 9.56
Kpinnou	60.00 ± 20.00	0.00 ± 0.00	66.67 ± 33.33	42.22 ± 17.56
Mean host species infestation rate (%)	73.23 ± 2.81	23.61 ± 7.28	33.33 ± 11.43	

**Table 5:** Genotypic frequencies of homozygous *Bulinus globosus* individuals from the digestion of the *Cox1* and *SSU* genes with the restriction endonucleases (*DdeI*, *HaeIII*, and *HinfI*).

Locality	Species	Sample size	Genotypic frequencies (%)									*p*value (H‐W)
			***Cox1*, *SSU DdeI***			***Cox1*, *SSU HinfI***			***Cox1*, *SSU HaeIII***			
			**A1A1**	**A1A2**	**A2A2**	**B1B1**	**B1B2**	**B2B2**	**C1C1**	**C1C2**	**C2C2**	
Sô‐Ava	*B. globosus*	20	6 (30)	3 (15)	11 (55)	2 (10)	3 (15)	15 (75)	3 (15)	16 (80)	1 (5)	0.001
Aplahoué	*B. globosus*	10	2 (20)	2 (20)	6 (60)	1 (10)	8 (80)	1 (10)	2 (20)	1 (10)	7 (70)	0.001
Houéyogbé	*B. globosus*	12	1 (8.33)	10 (83.33)	1 (8.33)	1 (8.33)	3 (25)	8 (66.66)	3 (25)	8 (66.66)	1 (8.33)	0.001
Za‐Kpota	*B. globosus*	12	3 (25)	8 (66.66)	1 (8.33)	0	10 (83.33)	2 (16.66)	5 (41.66)	7 (58.33)	0	0.001
Kpinnou	*B. globosus*	10	3 (30)	2 (20)	5 (50)	4 (40)	1 (10)	5 (50)	6 (60)	0	4 (40)	0.001
Azowlissè	*B. globosus*	16	6 (37.5)	1 (6.25)	9 (56.25)	0	6 (37.5)	10 (62.5)	4 (25)	11 (68.5)	1 (6.25)	0.001

*Note:* A1A1, A2A2, B1B1, B2B2, C1C1, and C2C2: number of homozygous genotypes; A1A2, B1B2, and C1C2: number of heterozygous genotype.

**Table 6:** Genotypic frequencies of homozygous *Bulinus forskalii* individuals from the digestion of the *Cox1* and *SSU* genes with the restriction endonucleases (*DdeI*, *HaeIII*, and *HinfI*).

Locality	Species	Sample size	Genotypic frequencies (%)									*p*value (H‐W)
			***Cox1*, SSU *DdeI***			***Cox1*, SSU *HinfI***			***Cox1*, *SSU HaeIII***			
			**A1A1**	**A1A2**	**A2A2**	**B1B1**	**B1B2**	**B2B2**	**C1C1**	**C1C2**	**C2C2**	
Sô‐Ava	*B. forskalii*	2	0	1 (50)	1 (50)	0	1 (50)	1 (50)	0	2 (100)	0	0.001
Azowlissè	*B. forskalii*	1	1 (100)	0	0	1 (100)	0	0	0	1 (100)	0	0.001
Houéyogbé	*B. forskalii*	1	0	1 (100)	0	0	0	1 (100)	1 (100)	0	0	0.001
Kpinnou	*B. forskalii*	2	1 (50)	1 (50)	0	0	0	2 (100)	2 (100)	0	0	0.001

*Note:* A1A1, A2A2, B1B1, B2B2, C1C1, and C2C2: number of homozygous genotypes; A1A2, B1B2, and C1C2: number of heterozygous genotype.

**Table 7:** Genotypic frequencies of homozygous *Bulinus truncatus* individuals from the digestion of the *Cox1 and SSU* genes with the restriction endonucleases (*DdeI*, *HaeIII*, and *HinfI*).

Locality	Species	Sample size	Genotypic frequencies (%)									*p*value (H‐W)
			***Cox1 SSU*, *DdeI***			***Cox1 SSU*, *HinfI***			***Cox1 SSU*, *HaeIII***			
			**A1A1**	**A1A2**	**A2A2**	**B1B1**	**B1B2**	**B2B2**	**C1C1**	**C1C2**	**C2C2**	
Sô‐Ava	*B. truncatus*	6	2 (33.3)	1 (16.66)	3 (50)	1 (16.66)	1 (16.66)	4 (66.66)	5 (83.33)	0	1 (16.66	0.001
Azowlissè	*B. truncatus*	4	1 (25)	2 (50)	1 (25)	2 (50)	0	2 (50)	0	1	3 (75)	0.001

*Note:* A1A1, A2A2, B1B1, B2B2, C1C1, and C2C2: number of homozygous genotypes; A1A2, B1B2, and C1C2: number of heterozygous genotype.

**Table 8:** *H*_o_, *H*_e_, *FIS*, and *FST* values in *Bulinus globosus* populations using the molecular markers *Cox1* and *SSU* with enzymatic digestion by *HinfI*, *HaeIII*, and *DdeI*.

Genes	*Cox1 HinfI*, *HaeIII*, *DdeI*					*SSU HinfI*, *HaeIII*, *DdeI*				
Locality	Species	*H*_*o*_	*H*_*e*_	*FIS*	*FST*	Species	*H*_*o*_	*H*_*e*_	*FIS*	*FST*
Sô‐Ava	*B. globosus*	0.01	0.20	0.90	0.05	*B. globosus*	0.01	0.19	0.85	0.09
Aplahoué	*B. globosus*	0.02	0.19	0.81	0.09	*B. globosus*	0.02	0.18	0.76	0.14
Houéyogbé	*B. globosus*	0.02	0.18	0.76	0.14	*B. globosus*	0.02	0.17	0.71	0.19
Za‐Kpota	*B. globosus*	0.02	0.19	0.81	0.09	*B. globosus*	0.02	0.16	0.66	0.23
Kpinnou	*B. globosus*	0.01	0.17	0.76	0.19	*B. globosus*	0.01	0.20	0.90	0.05
Azowlissè	*B. globosus*	0.01	0.16	0.71	0.23	*B. globosus*	0.01	0.17	0.76	0.19

**Table 9:** Values of *H*_o_, *H*_e_, *FIS*, and *FST* in the populations of *Bulinus truncatus* using the molecular markers *Cox I* and *SSU* through enzymatic digestion with *HinfI*, *HaeIII*, and *DdeI*.

Genes	*Cox1 Hinf*I, *HaeIII*, *DdeI*					*SSU Hinf*I, *HaeIII*, *DdeI*				
Locality	Species	*H*_*o*_	*H*_*e*_	*FIS*	*FST*	Species	*H*_*o*_	*H*_*e*_	*FIS*	*FST*
Sô‐Ava	*B. truncatus*	0.01	0.20	0.90	0.05	*B. truncatus*	0.02	0.19	0.80	0.09
Azowlissè	*B. truncatus*	0.02	0.19	0.80	0.09	*B. truncatus*	0.01	0.20	0.90	0.05

**Table 10:** Values of *H*_o_, *H*_e_, *FIS*, and *FST* in the populations of *Bulinus forskalii* with molecular markers *Cox I* and *SSU* through enzymatic digestion with *HinfI*, *HaeIII*, and *DdeI*.

Genes	*Cox1 HinfI*, *HaeIII*, *DdeI*					*SSU HinfI, HaeIII*, *DdeI*				
Locality	Species	*H*_*o*_	*H*_*e*_	*FIS*	*FST*	Species	*H*_*o*_	*H*_*e*_	*FIS*	*FST*
Sô‐Ava	*B. forskalii*	0.02	0.20	0.85	0.05	*B. forskalii*	0.01	0.18	0.80	0.14
Azowlissè	*B. forskalii*	0.02	0.19	0.80	0.09	*B. forskalii*	0.02	0.20	0.85	0.05
Houéyogbé	*B. forskalii*	0.01	0.18	0.80	0.14	*B. forskalii*	0.01	0.17	0.76	0.19
Kpinnou	*B. forskalii*	0.02	0.16	0.66	0.23	*B. forskalii*	0.02	0.18	0.80	0.14

### 3.3. Infestation Rate of *Bulinus* Species

The nonparametric Kruskal–Wallis test indicated a significant difference (*p* = 0.01) in the number of *Bulinus* snails infested across the six localities. Overall, of the 338 *B. globosus* specimens collected and tested for cercarial release, 248 released cercariae, yielding an overall infestation rate of 73.37*%* ± 2.80*%*. However, this rate varied by locality: Houéyogbé (53.84*%* ± 18.84*%*), Kpinnou (60*%* ± 20*%*), Sô‐Ava (64.12*%* ± 4.6*%*), Azowlissè (76*%* ± 4.90*%*), Aplahoué (85.71*%* ± 6.39*%*), and Za‐Kpota (100*%* ± 8.90*%*). Of the 46 *B. forskalii* collected and tested, 34 were infested, resulting in an infestation rate of 73.91*%* ± 7.53*%*. Locality‐specific infestation rates were as follows: Sô‐Ava (66.67*%* ± 19.24*%*), Azowlissè (66.67*%* ± 19.24*%*), Kpinnou (66.67*%* ± 33.33*%*), and Houéyogbé (80*%* ± 8.94*%*). For *B. truncatus*, 17 of 23 specimens released schistosome cercariae, corresponding to an infestation rate of 73.91*%* ± 10.65*%*. Infestation was heterogeneous across the two localities where this species was found: Azowlissè (66.67*%* ± 33.33*%*) and Sô‐Ava (75.00*%* ± 11.18*%*). Overall, the most heavily infested localities were Azowlissè (69.78*%* ± 5.01*%*) and Sô‐Ava (68.6*%* ± 4.07*%*), followed by Houéyogbé (44.61*%* ± 9.56*%*), Kpinnou (42.22*%* ± 17.56*%*), Za‐Kpota (33.33*%* ± 10.54*%*), and Aplahoué (28.57*%* ± 8.24*%*) (Table 4).

### 3.4. Genotyping of *Bulinus* and Population Genotypic Structure

Restriction enzyme digestion of PCR‐amplified *Cox1* and *SSU* fragments provides a straightforward approach to assess polymorphism and infer population genetic structure.

**Figure 2:**
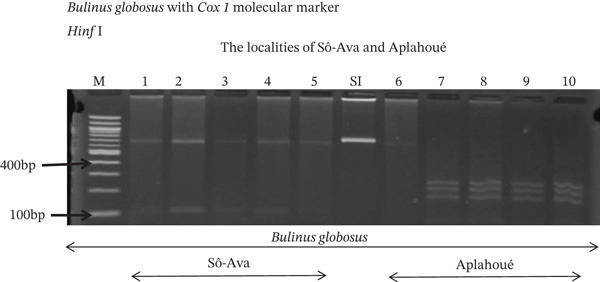
Enzymatic digestion of the *Cox1* gene of *Bulinus globosus* in the localities (Sô‐Ava and Aplahoué). Legend: M: marker (100 bp), SI: sequence of interest. 1, 2, 3, 4, 5 = *Bulinus globosus* (Sô‐Ava); 6, 7, 8, 9, 10 = *Bulinus globosus* (Aplahoué).

**Figure 3:**
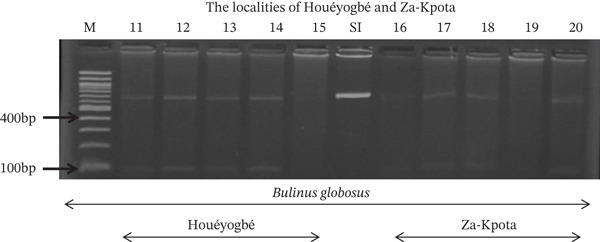
Enzymatic digestion of the *Cox1* gene of *Bulinus globosus* in the localities (Houéyogbé and Za‐Kpota). Legend: M: marker (100 bp), SI: sequence of interest. 11, 12, 13, 14, 15 = *Bulinus globosus* (Houéyogbé); 16, 17, 18, 19, 20 = *Bulinus globosus* (Za‐Kpota).

**Figure 4:**
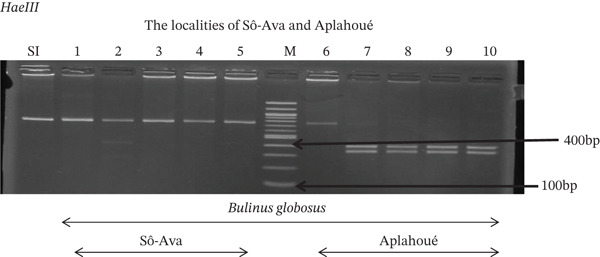
Enzymatic digestion of the *Cox1* gene of *Bulinus globosus* in the localities (Sô‐Ava and Aplahoué). Legend: M: marker (100 bp), SI: sequence of interest. 1, 2, 3, 4, 5 = *Bulinus globosus* (Sô‐Ava); 6, 7, 8, 9, 10 = *Bulinus globosus* (Aplahoué).

**Figure 5:**
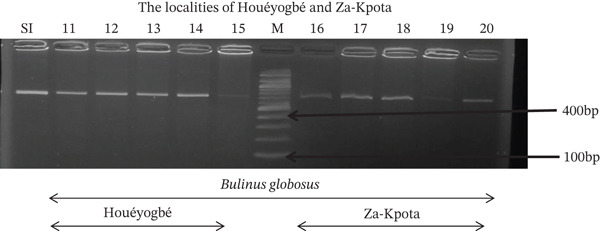
Enzymatic digestion of the *Cox1* gene of *Bulinus globosus* in the localities (Houéyogbé and Za‐Kpota). Legend: M: marker (100 bp), SI: sequence of interest. 11, 12, 13, 14, 15 = *Bulinus globosus* (Houéyogbé); 16, 17, 18, 19, 20 = *Bulinus globosus* (Za‐Kpota).

**Figure 6:**
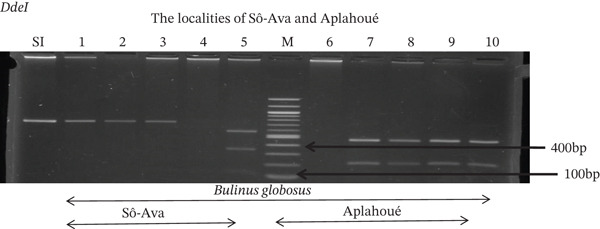
Enzymatic digestion of the *Cox1* gene of *Bulinus globosus* in the localities (Sô‐Ava and Aplahoué). Legend: M: marker (100 bp), SI: sequence of interest. 1, 2, 3, 4, 5 = *Bulinus globosus* (Sô‐Ava); 6, 7, 8, 9, 10 = *Bulinus globosus* (Aplahoué).

**Figure 7:**
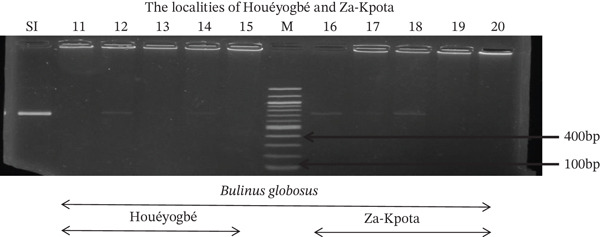
Enzymatic digestion of the *Cox1* gene of *Bulinus globosus* in the localities (Houéyogbé and Za‐Kpota). Legend: M: marker (100 bp), SI: sequence of interest. 11, 12, 13, 14, 15 = *Bulinus globosus* (Houéyogbé); 16, 17, 18, 19, 20 = *Bulinus globosus* (Za‐Kpota).

**Figure 8:**
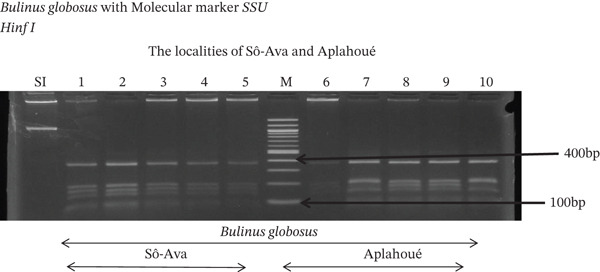
Enzymatic digestion of the *SSU* gene of *Bulinus globosus* in the localities (Sô‐Ava and Aplahoué). Legend: M: marker (100 bp), SI: sequence of interest. 1, 2, 3, 4, 5 = *Bulinus globosus* (Sô‐Ava); 6, 7, 8, 9, 10 = *Bulinus globosus* (Aplahoué).

**Figure 9:**
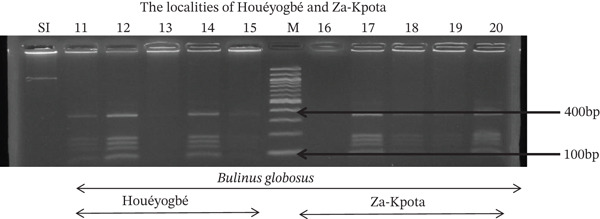
Enzymatic digestion of the *SSU* gene of *Bulinus globosus* in the localities (Houéyogbé and Za‐Kpota). Legend: M: marker (100 bp), SI: sequence of interest. 11, 12, 13, 14, 15 = *Bulinus globosus* (Houéyogbé); 16, 17, 18, 19, 20 = *Bulinus globosus* (Za‐Kpota).

**Figure 10:**
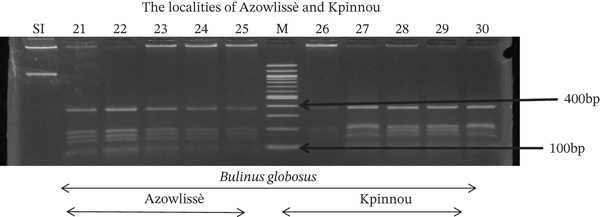
Enzymatic digestion of the *SSU* gene of *Bulinus globosus* in the localities (Azowlissè and Kpinnou). Legend: M: marker (100 bp), SI: sequence of interest. 21, 22, 23, 24, 25 = *Bulinus globosus* (Azowlissè); 26, 27, 28, 29, 30 = *Bulinus globosus* (Kpinnou).

**Figure 11:**
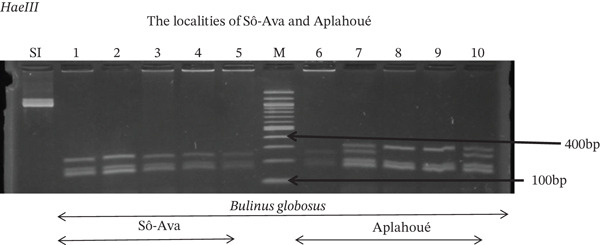
Enzymatic digestion of the *SSU* gene of *Bulinus globosus* in the localities (Sô‐Ava and Aplahoué). Legend: M: marker (100 bp), SI: sequence of interest. 1, 2, 3, 4, 5 = *Bulinus globosus* (Sô‐Ava); 6, 7, 8, 9, 10 = *Bulinus globosus* (Aplahoué).

**Figure 12:**
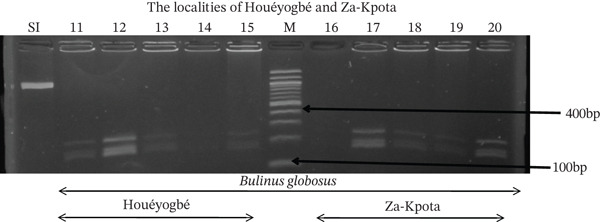
Enzymatic digestion of the *SSU* gene of *Bulinus globosus* in the localities (Houéyogbé and Za‐Kpota). Legend: M: marker (100 bp), SI: sequence of interest. 11, 12, 13, 14, 15 = *Bulinus globosus* (Houéyogbé); 16, 17, 18, 19, 20 = *Bulinus globosus* (Za‐Kpota).

**Figure 13:**
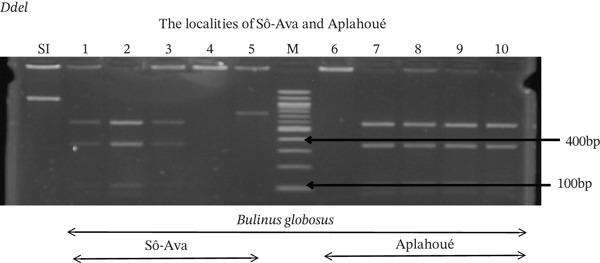
Enzymatic digestion of the *SSU* gene of *Bulinus globosus* in the localities (Sô‐Ava and Aplahoué). Legend: M: marker (100 bp), SI: sequence of interest. 1, 2, 3, 4, 5 = *Bulinus globosus* (Sô‐Ava); 6, 7, 8, 9, 10 = *Bulinus globosus* (Aplahoué).

**Figure 14:**
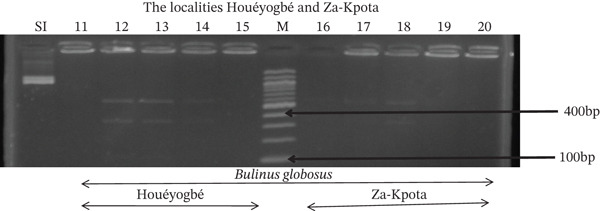
Enzymatic digestion of the *SSU* gene of *Bulinus globosus* in the localities (Houéyogbé and Za‐Kpota). Legend: M: marker (100 bp), SI: sequence of interest. 11, 12, 13, 14, 15 = *Bulinus globosus* (Houéyogbé); 16, 17, 18, 19, 20 = *Bulinus globosus* (Za‐Kpota).

**Figure 15:**
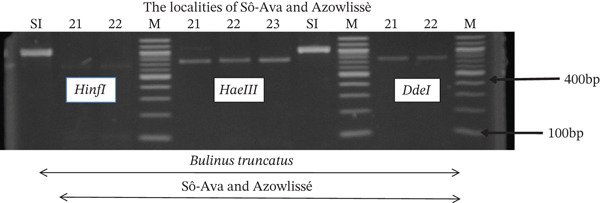
Enzymatic digestion of the *Cox1* gene from *Bulinus truncatus* in the localities (Sô‐Ava and Azowlissè). Legend: M: marker (100 bp), SI: sequence of interest. 21 = *Bulinus truncatus* (Sô‐Ava); 22 = *Bulinus truncatus* (Azowlissè); *HinfI*, *HaeIII*, and *DdeI* = restriction enzymes.

**Figure 16:**
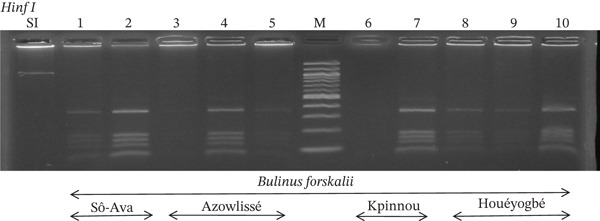
Enzymatic digestion of the *Cox1* gene of *Bulinus forskalii* in the localities (Sô‐Ava, Azowlissè, Kpinnou, and Houéyogbé). Legend: M: marker (100 bp), SI: sequence of interest. 1, 2 = *Bulinus forskalii* (Sô‐Ava); 3, 4, 5 = *Bulinus forskalii* (Azowlissè); 6, 7 = *Bulinus forskalii* (Kpinnou); 8, 9, 10 = *Bulinus forskalii* (Houéyogbé).

**Figure 17:**
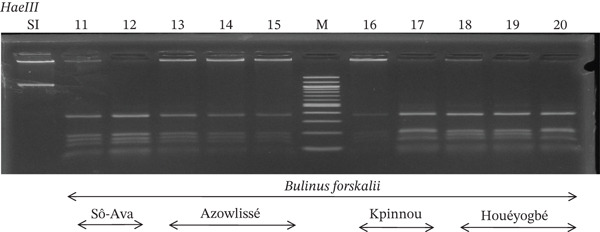
Enzymatic digestion of the *Cox1* gene of *Bulinus forskalii* in the localities (Sô‐Ava, Azowlissè, Kpinnou, and Houéyogbé). Legend: M: marker (100 bp), SI: sequence of interest. 11, 12 = *Bulinus forskalii* (Sô‐Ava); 13, 14, 15 = *Bulinus forskalii* (Azowlissè); 16, 17 = *Bulinus forskalii* (Kpinnou); 18, 19, 20 = *Bulinus forskalii* (Houéyogbé).

**Figure 18:**
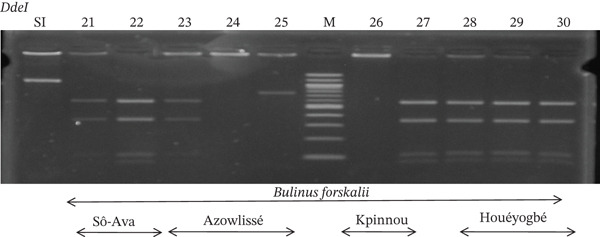
Enzymatic digestion of the *Cox1* gene of *Bulinus forskalii* in the localities (Sô‐Ava, Azowlissè, Kpinnou, and Houéyogbé). Legend: M: marker (100 bp), SI: sequence of interest. 21, 22 = *Bulinus forskalii* (Sô‐Ava); 23, 24, 25 = *Bulinus forskalii* (Azowlissè); 26, 27 = *Bulinus forskalii* (Kpinnou); 28, 29, 30 = *Bulinus forskalii* (Houéyogbé).

**Figure 19:**
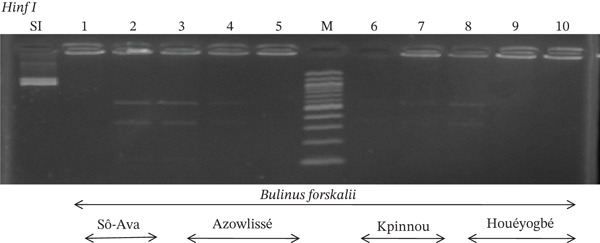
Enzymatic digestion of the *Cox1* gene of *Bulinus forskalii* in the localities (Sô‐Ava, Azowlissè, Kpinnou, and Houéyogbé). Legend: M: marker (100 bp), SI: sequence of interest. 1, 2 = *Bulinus forskalii* (Sô‐Ava); 3, 4, 5 = *Bulinus forskalii* (Azowlissè); 6, 7 = *Bulinus forskalii* (Kpinnou); 8, 9, 10 = *Bulinus forskalii* (Houéyogbé).

**Figure 20:**
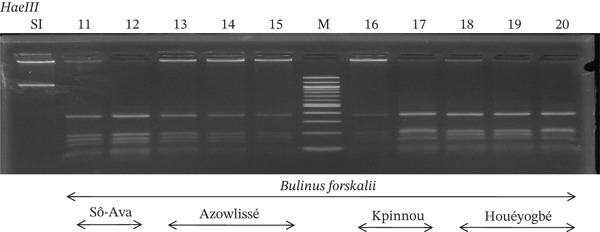
Enzymatic digestion of the *Cox1* gene of *Bulinus forskalii* in the localities (Sô‐Ava, Azowlissè, Kpinnou, and Houéyogbé). Legend: M: marker (100 bp), SI: sequence of interest. 11, 12 = *Bulinus forskalii* (Sô‐Ava); 13, 14, 15 = *Bulinus forskalii* (Azowlissè); 16, 17 = *Bulinus forskalii* (Kpinnou); 18, 19, 20 = *Bulinus forskalii* (Houéyogbé).

**Figure 21:**
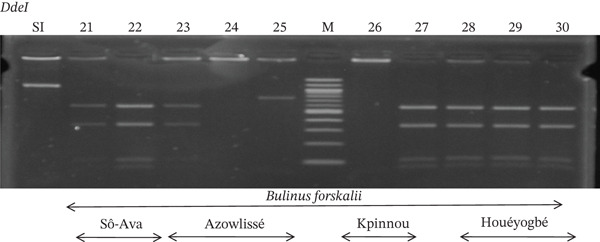
Enzymatic digestion of the *Cox1* gene of *Bulinus forskalii* in the localities (Sô‐Ava, Azowlissè, Kpinnou, and Houéyogbé). Legend: M: marker (100 bp), SI: sequence of interest. 21, 22 = *Bulinus forskalii* (Sô‐Ava); 23, 24, 25 = *Bulinus forskalii* (Azowlissè); 26, 27 = *Bulinus forskalii* (Kpinnou); 28, 29, 30 = *Bulinus forskalii* (Houéyogbé).

Tables 5, 6, and 7 present the genotypic frequencies across different digestions. Analysis revealed that *Cox1* digestions (*DdeI*, *HaeIII*, and *HinfI*) produced predominantly homozygous individuals in all populations, ranging from 62.5% in Azowlissè to 100% in Kpinnou, with heterozygotes observed at low frequencies (Figures 2, 3, 4, 5, and 6). In contrast, SSU digestions exhibited higher heterozygosity, with heterozygous individuals reaching 43.75% in Azowlissè and up to 83.33% in Houéyogbé and Za‐Kpota, while homozygotes were less frequent (10% in Sô‐Ava to 56.75% in Azowlissè) (Figures 7, 8, 9, 10, 11, 12, 13, 14, and 15). None of the populations was in HWE (*p* < 10^−3^), indicating deviations from random mating and suggesting population substructuring. Overall, the *SSU* marker demonstrated higher levels of molecular diversity than *Cox1* in *B. globosus* populations. Analysis of *Cox1* and *SSU* fragments digested with *DdeI*, *HaeIII*, and *HinfI* revealed that *Cox1* digestions produced predominantly homozygous individuals, with high frequencies in Sô‐Ava (100%) and moderate frequencies in Azowlissè (50%), whereas heterozygotes were rare (Figures 16, 17, 18, 19, 20, and 21). In contrast, *SSU* digestions showed higher heterozygosity, with heterozygous individuals predominating in both Sô‐Ava and Azowlissè, and lower homozygous frequencies across populations. None of the populations was in HWE (*p* < 0.05), indicating deviations from random mating and suggesting population substructuring. Overall, the *SSU* marker exhibited greater molecular diversity than *Cox1* in *B. truncatus* populations. Analysis of *Cox1* and *SSU* fragments digested with *DdeI*, HaeIII, and *HinfI* showed relatively high genotypic frequencies for both homozygous and heterozygous individuals across all populations. None of the populations was in HWE (*p* < 0.05), indicating deviations from random mating and potential population substructuring. Across all three *Bulinus* species, the *SSU* marker consistently exhibited higher heterozygosity and greater molecular diversity than *Cox1*, suggesting it is a more informative marker for assessing genetic variability in these snails.

### 3.5. Molecular Diversity and Deviation From Panmixia

#### 3.5.1. *B. globosus*

Analysis of HinfI digested *Cox1* and *SSU* markers showed consistently low (*H*_o_ = 0.01–0.02) relative to (*H*_e_ = 0.16–0.20) across all populations. High *FIS* indicated significant deviations from HWE and a notable heterozygote deficit. Genetic differentiation among populations (*FST*) ranged from 0.05 to 0.23. Moderate differentiation (0.05 < *FST* < 0.15) was observed in Sô‐Ava, Houéyogbé, Kpinnou, Aplahoué, and Za‐Kpota, while stronger differentiation (0.15 < *FST* < 0.25) occurred in Azowlissè (*Cox1*) and in Houéyogbé and Za‐Kpota (*SSU*) (Table 8). These results indicate restricted gene flow and potential inbreeding, likely reflecting ecological isolation and population fragmentation across the study sites. Analysis of PCR‐RFLP data revealed low (*H*_o_ = 0.01–0.02) across all populations, whereas *H*_e_ ranged from 0.14 to 0.20, indicating a consistent heterozygote deficit. Significant deviations from HWE were detected at all sites, and high *FIS* suggested restricted gene flow and potential inbreeding. Genetic differentiation among populations, measured by *FST*, was generally moderate (0.05 < *FST* < 0.15) in Sô‐Ava, Kpinnou, Aplahoué, Houéyogbé, and Azowlissè, but higher (0.15 < *FST* < 0.25) in Houéyogbé and Azowlissè, and very high (*FST* > 0.25) in Za‐Kpota. These patterns were consistent across analyses using both *Cox1* and *SSU* markers with *HaeIII* and *DdeI* endonucleases (Table 8). Overall, the data indicate structured *Bulinus* populations with limited gene flow, moderate to high differentiation depending on the locality, and widespread deviations from HWE, reflecting potential inbreeding or substructuring.

#### 3.5.2. *B. truncatus*

Across all analyses using *Cox1* and *SSU* markers digested with *HinfI*, *HaeIII*, or *DdeI*, *H*_o_ in *B. truncatus* populations was consistently low (0.01–0.02), whereas *H*_e_ ranged from 0.19 to 0.20, indicating a pronounced heterozygote deficit. Moderate genetic differentiation was observed between the two populations studied (Sô‐Ava and Azowlissè; *F* *S* *T* = 0.05–0.09) (Table 9). Statistically significant deviations from HWE and consistently high *FIS* were recorded, reflecting restricted gene flow and potential inbreeding across these populations. These patterns were consistent across all enzyme marker combinations, confirming reduced genetic variability and population structuring in *B. truncatus*.

#### 3.5.3. *B. forskalii*

Across all analyses using *Cox1* and *SSU* markers with *HaeIII*, *HinfI*, or *DdeI* digestion, *B. forskalii* populations exhibited consistently low (*H*_o_ = 0.01–0.02) relative to (*H*_e_ = 0.17–0.20), indicating a pronounced heterozygote deficit. Genetic differentiation among populations (*FST*) ranged from 0.05 to 0.25. Moderate differentiation (0.05 < *FST* < 0.15) was observed in Sô‐Ava, Kpinnou, Azowlissè, and Houéyogbé, while higher differentiation (0.15 < *FST* < 0.25) occurred in Houéyogbé and Kpinnou depending on the enzyme–marker combination (Table 10). Statistically significant deviations from HWE were detected at all sites, and high *FIS* confirmed restricted gene flow and potential inbreeding across populations. These patterns were consistent across all enzyme marker analyses, reflecting structured populations with limited genetic connectivity.

Across all *Bulinus* species analyzed using *Cox1* and *SSU* markers digested with *HaeIII, HinfI*, or *DdeI*, *H*_o_ was consistently low (0.01–0.02), whereas *H*_e_ ranged from 0.14 to 0.20, indicating a pronounced heterozygote deficit. Deviations from HWE were statistically significant across all populations, and high *FIS* confirmed restricted gene flow and potential inbreeding. Genetic differentiation (*FST*) varied among localities, generally showing moderate differentiation (0.05 < *FST* < 0.15) in populations from Sô‐Ava, Kpinnou, Aplahoué, Houéyogbé, and Azowlissè, while higher differentiation (0.15 < *FST* < 0.25) was observed in certain localities such as Houéyogbé, Kpinnou, Azowlissè, and Za‐Kpota depending on species and enzyme marker combination. These results consistently indicate structured *Bulinus* populations with limited genetic connectivity, likely driven by ecological isolation, population fragmentation, and localized gene flow restrictions across the study sites.

## 4. Discussion

The abundance of *Bulinus* populations varied among the sampled localities. *B. globosus* was present in all study sites, showing the highest density in Sô‐Ava. This species coexisted sympatrically with *B. truncatus* in other sampling sites, where distinguishing the two populations was often difficult due to overlapping ecological niches and the limitations of morphological identification. Similar observations were reported on the shores of Lake Niger [30]. *B. truncatus* was highly abundant only in two localities, Sô‐Ava and Aplahoué. Several studies have previously emphasized the high morbidity associated with urinary schistosomiasis in Africa, mainly due to the continuous presence of *Bulinus* intermediate hosts that facilitate *Schistosoma* transmission [4]. *B. globosus* was found to occupy transitional habitats where water availability fluctuates substantially between seasons, which may explain its relative abundance across most sites. This adaptability reflects its broad tolerance to both biotic and abiotic conditions. Conversely, the restricted distribution of *B. truncatus* (observed only in Sô‐Ava and Azowlissè) likely reflects unsuitable ecological conditions in other environments, including unfavorable pH, salinity, and oxygen levels. In addition to ecological constraints, reproductive biology particularly frequent inbreeding combined with environmental fluctuations likely generated bottlenecks that reduced the effective population size of *B. truncatus* [15]. The genetic structure of *Bulinus* populations revealed deviations from panmixia in Sô‐Ava, Azowlissè, Aplahoué, Houéyogbé, and Za‐Kpota, characterized by heterozygote deficits. Deviations from HWE may arise from the Wahlund effect [31], inbreeding, null alleles [32], genetic drift, or selection against heterozygotes. Given the molecular methods used, null alleles can be excluded. Thus, the observed deviations likely result from inbreeding, restricted dispersal, or population subdivision, although genetic drift and the Wahlund effect cannot be ruled out. The *H*_*o*_ deficits and deviations from panmixia indicate nonrandom mating and limited gene flow, reflecting underlying biological and demographic processes that shape *Bulinus* genetic diversity. These findings underscore the need to consider population structure, effective population size, and reproductive behavior when interpreting genetic indices and assessing adaptive potential. Comparable results were reported in *Bulinus* populations from Zimbabwe [33], Morocco [34], and Madagascar [35, 36], as well as in other self‐fertilizing gastropods such as *Galba trunculata* [4], *Biomphalaria pfeifferi* [35], and *B. forskalii* [15]. The calculated *FST* values revealed several degrees of local genetic differentiation, suggesting reduced gene flow among populations of each species. Such structuring may result from limited dispersal, reproductive behavior, demographic instability, and the metapopulation dynamics of *Bulinus*. Extinction recolonization processes are known to strongly influence genetic differentiation in this genus [37], while gene flow may also be shaped by the mobility of snails and human population movements [38]. The molecular diversity analyses using RFLP of *Cox1* and *SSU* genes with *HaeIII*, *HinfI*, and *DdeI* revealed substantial polymorphism, particularly within *B. globosus* populations. This diversity may reflect reproductive strategies, migration, and the species′ remarkable ecological adaptability [33]. The observed levels of polymorphism are consistent with findings in *B. globosus* populations from Zimbabwe [33]. Similar patterns were detected in *B. truncatus* from Sô‐Ava and Azowlissè, suggesting that these genetic markers are stable across both species in Benin. Microsatellite markers have been recognized as powerful tools for further exploring such genetic variation, although their effectiveness depends on careful primer selection for *Bulinus*. The higher abundance and genetic structuring of *B. globosus* may reflect an earlier adaptive radiation followed by dispersal, while the ecological similarity between *B. globosus* and *B. truncatus* may be driven by selective advantages acquired during coevolution with *S. haematobium* [39]. Both species thus represent key intermediate hosts in the parasite′s transmission cycle. Recent studies in Benin particularly [10] on water quality and snail ecology in the Sô‐Ava region and [11] on urinary schistosomiasis and geohelminthiasis among schoolchildren have provided valuable ecological insights. Nevertheless, molecular investigations remain scarce, emphasizing the need for extended research. Sequencing of candidate genes, including microsatellites and other hypervariable markers, is recommended to deepen understanding of *Bulinus* population dynamics. Molecular analyses revealed variable genetic diversity across southern Benin populations, including heterozygosity deficits, deviations from panmixia, and monomorphic populations. These results are critical for understanding *S. haematobium* transmission and designing targeted control strategies. Monomorphic populations such as *B. globosus* at Za‐Kpota and Houéyogbé and *B. truncatus* at Sô‐Ava and Azowlissè may be more susceptible to uniform molluscicidal treatment. Based on previous findings [40], the plant *Talinum triangulare*, an effective and locally available molluscicide in Nigeria, represents a promising option for Benin. Populations with moderate polymorphism, such as *B. globosus* in Sô‐Ava and Aplahoué, may require repeated or combined interventions. Application of *Talinum* in high‐density snail habitats, coupled with environmental management, could effectively reduce intermediate host abundance and interrupt *S. haematobium* transmission. Continuous molecular monitoring using *Cox1* and *SSU* markers would enable evaluation of intervention efficacy and adaptation of control measures according to the genetic structure of each population. Integrating molecular insights with plant‐based molluscicides offers a sustainable, targeted, and environmentally friendly approach for urinary schistosomiasis control in Benin. These findings have significant implications for schistosomiasis surveillance and control strategies in Benin and across West Africa. The observed genetic structuring and deviations from panmixia within *Bulinus* populations suggest that transmission foci of *S. haematobium* are likely maintained by locally adapted and partially isolated snail populations. This underlines the need for geographically tailored surveillance and control programs rather than uniform interventions. Regular molecular monitoring of *Bulinus* populations using mitochondrial and ribosomal markers (*Cox1* and *SSU*), as well as more sensitive genomic tools such as microsatellites or next‐generation sequencing (NGS)–based approaches, will be essential for detecting shifts in genetic composition that could influence parasite transmission dynamics. Future studies should prioritize high‐resolution sequencing of candidate genes to unravel fine‐scale population connectivity and adaptive processes within *Bulinus* species. Integrating genomic data with ecological, epidemiological, and spatial analyses will provide a robust framework for predicting transmission risks and optimizing schistosomiasis control. Such molecular approaches, combined with environmentally sustainable interventions like the use of *Talinum triangulare*, offer promising avenues for long‐term, evidence‐based elimination programs.

## 5. Conclusions

This study provides the first scientific insights, albeit preliminary, into the genetic structure and diversity of *Bulinus* populations in Benin. Across all species, a significant heterozygote deficit was observed, likely attributable to reproductive patterns, sympatric co‐occurrence with morphological misidentification, and genetic drift. The application of PCR‐RFLP and restriction enzyme analysis revealed the first molecular polymorphisms in these snail populations. These findings have important implications for schistosomiasis surveillance, as they highlight the potential for certain populations to sustain parasite transmission. Given the recorded abundance of *Bulinus* in highly endemic areas, continued monitoring is essential. Future studies employing high‐resolution sequencing approaches are strongly recommended to more precisely characterize genetic diversity, population structure, and the potential for transmission of *S. haematobium*. Such data will provide a robust basis for evaluating and optimizing control strategies, including targeted snail management and public health interventions, thereby contributing to more effective disease prevention in Benin.

## Author Contributions

Conceptualization: Elokou Alabi; methodology: Elokou Alabi; validation: Michel Sezonlin; formal analysis: Jonathan Nougbode; investigation: Elokou Alabi, Jonathan Nougbode, Aboudou Chabi Do, Halfane Lehmane, and Paulin Sedah; resources: Elokou Alabi and Jonathan Nougbode; data curation: Elokou Alabi and Jonathan Nougbode; writing—original draft: Elokou Alabi; writing—review and editing: Halfane Lehmane, Haziz Sina, Adolphe Adjanohoun, Michel Sezonlin, and Lamine Baba‐Moussa; visualization: Elokou Alabi and Jonathan Nougbode; supervision: Michel Sezonlin and Lamine Baba‐Moussa.

## Funding

No funding was received for this manuscript.

## Conflicts of Interest

The authors declare no conflicts of interest.

## Data Availability

The data that support the findings of this study are available from the corresponding author upon reasonable request.
