# Correction to “Genetic Diversity and Ecological Distribution of *Bulinus* Populations in Southern Benin Using PCR‐RFLP Analysis”

**DOI:** 10.1155/bmri/9869213

**Published:** 2026-09-18

E. Alabi, A. C. Do, J. Nougbode, et al., “Genetic Diversity and Ecological Distribution of *Bulinus* Populations in Southern Benin Using PCR‐RFLP Analysis,” BioMed Research International no. 1 (2026): 5536391, https://doi.org/10.1155/bmri/5536391.

In the Methodology Section, Chapter 2.4, Statistical Analyses, References [28, 29] were included in error. The authors did not present the formulas used for the calculation of the genetic diversity parameters. The correct references are [42, 43].

We apologize for this error.

